# Analgesic and adjuvant co‐prescribing in Australian and Finnish residential care homes

**DOI:** 10.1111/ajag.70062

**Published:** 2025-07-01

**Authors:** Laura A. Dowd, Kaisu H. Pitkälä, Agathe D. Jadczak, Hanna‐Maria Roitto, Ulla L. Aalto, Riitta K.T. Saarela, Renuka Visvanathan, Shin J. Liau, Amanda J. Cross, J. Simon Bell

**Affiliations:** ^1^ Centre for Medicine Use and Safety (CMUS), Faculty of Pharmacy and Pharmaceutical Sciences Monash University Parkville Victoria Australia; ^2^ Department of General Practice and Primary Health Care University of Helsinki Helsinki Finland; ^3^ Unit of Primary Health Care Helsinki University Hospital Helsinki Finland; ^4^ Adelaide Geriatrics Training and Research with Aged Care (GTRAC) Centre, Adelaide Medical School, Faculty of Health and Medical Sciences University of Adelaide Adelaide South Australia Australia; ^5^ The Basil Hetzel Institute, The Queen Elizabeth Hospital Central Adelaide Local Health Network Adelaide South Australia Australia; ^6^ Department of Geriatrics University of Helsinki and Helsinki University Hospital Helsinki Finland; ^7^ Social Services, Health Care and Rescue Services Division, Oral Health Care City of Helsinki Helsinki Finland; ^8^ Aged and Extended Care Services, The Queen Elizabeth Hospital Central Adelaide Local Health Network Adelaide South Australia Australia; ^9^ Kuopio Research Centre of Geriatric Care University of Eastern Finland Kuopio Finland

**Keywords:** analgesics, nursing Homes, pain Management, polypharmacy

## Abstract

**Objective:**

To explore the co‐prescribing of analgesic and/or adjuvant medications among residents of Australian and Finnish residential care homes.

**Method:**

Secondary cross‐sectional analyses of prescribing data from residents of 12 Australian residential care homes in 2019 and 53 Finnish nursing homes and assisted living facilities in 2017–2018. Demographic characteristics and medication data were extracted from medical records and medication administration charts. Co‐prescribing was defined as more than one analgesic (acetaminophen, non‐steroidal anti‐inflammatory drugs and opioids) and/or adjuvant (gabapentinoids, tricyclic antidepressants and duloxetine) medication prescribed for regular administration.

**Results:**

Overall, 550 Australian residents (89 [IQR 84–92] years; 73% females) and 2423 Finnish residents (84 [IQR 65–103] years; 74% females) were included. Of 416 Australian residents prescribed any regular analgesic or adjuvant, 181 (44%) were prescribed two or more, including 66 (16%) who were prescribed three or more. Of 1406 Finnish residents prescribed any regular analgesic or adjuvant, 469 (33%) were prescribed two or more, including 87 (6%) who were prescribed three or more. Acetaminophen was co‐prescribed to more than 75% of Australian and 43% of Finnish residents prescribed other analgesics or adjuvants. Of 61 Australian residents and 186 Finnish residents prescribed gabapentinoids, 38 (62%) and 86 (46%) were co‐prescribed opioids. Opioids were co‐prescribed to 59%–83% of Australian and 25%–46% of Finnish residents prescribed adjuvants.

**Conclusions:**

Analgesic and adjuvant co‐prescribing was more prevalent for Australian than Finnish residents, which was largely driven by acetaminophen co‐prescribing to more than three quarters of analgesic or adjuvant users in Australia. Central nervous system‐active polypharmacy arising from high rates of adjuvant and opioid co‐prescribing warrants further attention.

**Policy impact**

The findings highlight the continued need for an increased awareness of analgesic and adjuvant co‐prescribing, particularly regarding central nervous system‐active polypharmacy. Policies should continue to focus on implementing regular analgesic stewardship interventions, including medication reviews, to ensure safe and effective medication use for older adults living in residental care homes.

## 1 INTRODUCTION

Australia and Finland are two countries with ageing populations. Overall, 17% of Australian and 23% of Finnish people were aged 65 years or older in 2021.^1^, ^2^ Pain management in residential care homes remains a significant challenge for health‐care providers, where 61% of 383 Australian and 57% of 5761 Finnish residents self‐report experiencing pain.^3^, ^4^ Given the high prevalence and complexity of pain management in older adults, clinicians often prescribe more than one traditional analgesic (i.e. acetaminophen, non‐steroidal anti‐inflammatory drugs [NSAIDs] and opioids) or adjuvant medications (i.e. gabapentinoids, tricyclic antidepressants [TCAs] and duloxetine) to provide comprehensive analgesia, commonly referred to as multimodal analgesia.^5^ The World Health Organization (WHO) analgesic ladder outlines a stepwise approach to pain management, recommending non‐opioid analgesics, such as acetaminophen or NSAIDs, as first‐line therapy, with the addition of opioids and adjuvant medications based on the severity and persistence of pain.^6^ Multimodal analgesia is increasingly utilised in non‐surgical settings to reduce adverse drug events (ADEs) through using lower doses of individual medications.^7^ Despite the potential benefits of multimodal analgesia, this practice risks exposing residents to polypharmacy and drug–drug interactions.^8^

Analgesic and adjuvant medications are an important component of overall pain management strategies, yet little is known about co‐prescribing patterns in residential care homes. As a result, research into the safety and efficacy of multimodal analgesia in residential care homes has been identified as a priority.^9^, ^10^, ^11^ Cross‐national comparisons of medication use are important to explore medication prescribing patterns and identify potential medication‐related issues.^12^ Comparing analgesic use in Australian and Finnish residential care homes is worthwhile because both countries have ageing populations and similar models of health‐care provision to residents.^13^, ^14^ Additionally, residents from both countries experience high rates of frailty, multimorbidity and polypharmacy, which brings challenges ensuring the safe and effective use of medications.^15^, ^16^ The objective of this study was to explore the co‐prescribing of analgesic and/or adjuvant medications among residents of Australian and Finnish residential care homes.

## 2 METHODS

### 2.1 Setting

This study investigated prescribing for residents of residential care homes (‘residential aged care services’ in Australia or ‘long‐term care facilities’ internationally). There are parallels between residential care homes in Australia and Finland. Residential care homes in both countries provide supported accommodation for older adults with long‐term care needs. In these settings, care is provided by nurses or care workers. Analgesics are predominantly charted by visiting general medical practitioners (GPs), dispensed by off‐site pharmacies and administered by nurses or care workers.^13^, ^14^ In Finland, there are nursing homes and assisted living facilities. Both provide 24‐hour care with a registered nurse in charge.^16^, ^17^ Assisted living facilities are designed to resemble a home‐like environment. The number of nursing home beds has declined and the number of assisted living facility beds increased over the past decade in Helsinki.^16^ A 2019 study reported residents living in Finnish nursing homes and assisted living facilities were similar in age, sex, number of comorbidities, dementia and number of medications.^18^

### 2.2 Design and Sample

We conducted secondary cross‐sectional analyses of medication prescribing data for residents from 12 residential care homes in South Australia in 2019 and 53 nursing homes or assisted living facilities in Helsinki in 2017–2018.

For the Australian sample, we analysed baseline data from the Frailty in Residential Sector over Time (FIRST) study.^19^ The FIRST study is a prospective 3‐year cohort study across 12 residential care homes in South Australia. In brief, all medically stable, permanent residents (i.e. living in the residential care homes for at least 8 weeks) were invited to participate. Residents considered to be at the end of life (<3 months to live) and those not able to complete baseline assessments in English were excluded. The Australian sample of residents was comparable to the average Australian residential care home population in terms of age (median: 89 [interquartile range, IQR: 84–92] vs. 87 [IQR: 84–95]) and sex (female: 73% vs. 66%).^20^

For the Finnish sample, we analysed medication prescribing data from residents living in nursing homes or assisted living facilities in Helsinki, Finland. All individuals aged 65 years or older living in nursing homes or assisted living facilities across all of Helsinki were eligible and invited to participate. Residents who had moderate‐to‐severe dementia (Clinical Dementia Rating [CDR] 2–3) and those not having a close proxy to give informed consent or refusals were excluded.^21^

### 2.3 Data collection

For the Australian sample, data were collected between March and October 2019 using a combination of resident medical records and observations, physical assessments and questionnaires administered by study nurses or site registered nurses. Researchers extracted medication data from medication charts. A full audit of each medication entry was completed. For the Finnish sample, data were collected by trained study nurses between 2017 and 2018 using resident medical records.

### 2.4 Resident characteristics

Demographic characteristics (age, sex) were obtained from resident medical records. For the Australian sample, observed pain was assessed using the Pain Assessment in Advanced Dementia (PAINAD) scale.^22^ Possible scores ranged from 0 to 10 and scores were classified into no (0) or mild/moderate (1–6) pain, as no residents were observed to be in severe pain (7–10). The study registered nurse observed each resident at rest for 5 min before applying the scale. The registered nurse was required to have known the respective resident for at least 2 weeks before conducting assessments. For the Finnish sample, self‐reported pain was assessed using the Health‐Related Quality of Life (HRQoL) 15D ‘discomfort and pain’ item.^23^ This item included symptoms of pain, ache, nausea and itching.

### 2.5 Medications

Traditional analgesics and adjuvant medications were defined by consensus discussion with the investigator team (File S1). Medications were categorised using the Anatomical Therapeutic Chemical (ATC) Classification System codes recommended by the WHO.^24^ Traditional analgesics included acetaminophen (i.e. paracetamol, ATC code: N02BE01), NSAIDs (M01A), non‐opioid plus opioid combinations (N02AJ) and opioids (N02A, including codeine preparations [R05DA04]). Adjuvant medications included gabapentinoids (N02BF), TCAs (N06AA) and selective serotonin and norepinephrine reuptake inhibitors [SNRIs] (duloxetine [N06AX21]). Medications prescribed for regular use were considered. This study did not address as‐needed (i.e. pro re nata, PRN) medications or self‐administration of any non‐prescription medications that were not recorded on the resident's medication chart.

### 2.6 Data analysis

Standardised data collection tables were developed and pilot tested with the investigator team (File S2). Analyses were conducted independently by investigators in their respective countries using these tables to avoid sharing of resident‐level data. Descriptive statistics were used to summarise demographic and clinical characteristics of residents. Aggregate medication data from both countries were compared and graphical representations were computed using Microsoft Excel version 365. Australian data were analysed using SAS, version 9.4 (SAS Institute, Inc., Cary, NC). Finnish data were analysed using Stata statistical software, version 16 (StataCorp, USA).

### 2.7 Ethical considerations

The Australian study was approved by the University of Adelaide Human Research Ethics Committee (HREC‐2018‐247) and was registered with the Monash University Human Research Ethics Committee (23620). The Finnish study was approved by ethics committee of Helsinki University Hospital (HUS/2042/2016).

## 3 RESULTS

### 3.1 Resident characteristics

A total of 2973 residents were included (*n* = 550 from Australia and *n* = 2423 from Finland [*n* = 750 nursing home residents and *n* = 1673 assisted living facility residents], Table 1). Australian and Finnish residents were comparable in age (median 89 [IQR 84–92] vs. median 84 [IQR 65–103]) and sex (females: 403 (73%) vs. 1791 (74%), Table 2). In total, 80 (15%) Australian residents were observed to be in any ‘mild/moderate pain’ using PAINAD. In total, 1677 (69%) Finnish residents self‐reported any ‘discomfort and pain’ and 431 (18%) self‐reported marked, severe or unbearable ‘discomfort and pain’ using 15D.

**TABLE 1:** Sample characteristics.

Country, state	Date of data collection	Sample size (*n*)	Number of facilities	Type of facilities	Inclusion/exclusion criteria	Method of medication data collection	Type of medication data
Australia, South Australia	March–October 2019	550	12	A not‐for‐profit aged care provider. Seven of the 12 services were located in the metropolitan area, two in the outer metropolitan area and three services were regional	Inclusion: all permanent residents living in the residential care homes for at least 8 weeks Exclusion: residents who were deemed to be medically unstable (e.g. experiencing delirium), have <3 months to live or not fluent in English	Trained study nurses scanned each resident's medication chart and researchers with pharmacy training extracted medication data. A full audit of each medication entry was completed for all residents	Prescribing of regular and PRN traditional analgesics and adjuvant medications Data on PRN administrations in the preceding 7 days were extracted
Finland, Helsinki	2017–2018	2423^a^	53	Nursing homes and assisted living facilities	Inclusion: all residents aged >65 years Exclusion: Those suffering moderate–severe dementia (Clinical Dementia Rating [CDR] 2–3) and not having a close proxy to give informed consent, refusals	Retrieved from medical records	Used regularly (by regular sequence)

^a^ Includes *n* = 750 nursing home residents and *n* = 1673 assisted living facility residents.

**TABLE 2:** Resident characteristics.

Country, state	Age (mean or median)	Female sex, *n* (%)	Pain prevalence, *n* (%)	Method of defining pain
Australia, South Australia	Median 89 (IQR 84–92)	403 (73)	*PAINAD* No pain: 470 (86) Mild/moderate pain: 80 (15)	The study nurse observed each resident for 5 min before completing the scale. Pain scores were classified into no (0) or mild/moderate (1–6) pain, as no residents were observed to be in severe pain (7–10)
Finland, Helsinki	Median 84 (IQR 65–103)	1791 (74)	*HRQoL 15D* ^a^ None: 733 (31) Mild: 1239 (52) Marked: 311 (13) Severe: 108 (5) Unbearable: 15 (1)	Self‐reported using the HRQoL 15D item ‘Discomfort and pain’ – Includes other symptoms of pain, ache, nausea and itching

Abbreviations: HRQoL, Health‐Related Quality of Life; IQR, interquartile range; PAINAD, Pain in Advanced Dementia.

^a^ Self‐reported pain prevalence available for 2406 residents.

### 3.2 Medication use

The prescribing of regular acetaminophen (380 (69%) vs. 1070 (44%)) and regular opioids (165 (30%) vs. 609 (25%)) was higher in the Australian sample than the Finnish sample. In both Australian and Finnish samples, the prescribing of regular NSAIDs was low (8 (2%) vs. 14 (1%)). The prescribing of regular gabapentinoids (61 (11%) vs. 186 (8%)) and regular TCAs (34 (6%) vs. 12 (1%)) was higher in the Australian sample than the Finnish sample. Duloxetine was prescribed to 12 (2%) Australian and 95 (4%) Finnish residents. The prescribing of analgesic and adjuvant medications was higher in Finnish nursing homes compared to Finnish assisted living facilities (acetaminophen 388 (53%) vs. 672 (40%), NSAIDs 6 (1%) vs. 8 (1%), opioids 233 (31%) vs. 117 (23%), gabapentinoids 69 (9%) vs. 117 (7%) and TCAs 5 (1%) vs. 7 (0%)), with the exception of duloxetine (29 (4%) vs. 66 (4%)).

### 3.3 Co‐prescribed combinations

**FIGURE 1:**
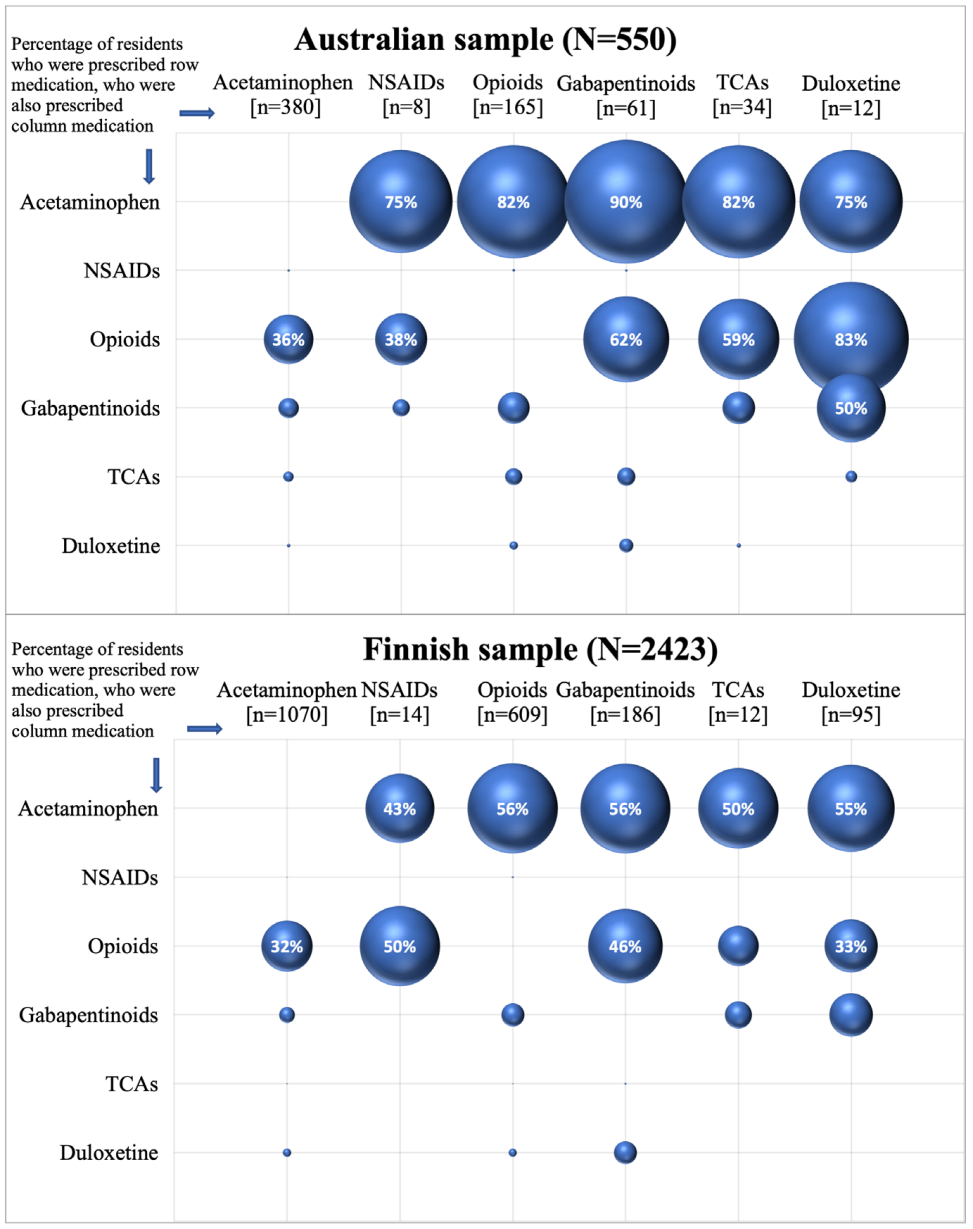
Co‐prescribing of regular analgesic and adjuvant medications in Australian and Finnish residents.

Of 416 Australian residents prescribed any regular analgesic or adjuvant medication, 181 (44%) were prescribed two or more, including 66 (16%) residents who were prescribed three or more (Table 3). Of 1406 Finnish residents prescribed any regular analgesic or adjuvant medication, 469 (33%) were prescribed two or more, including 87 (6%) residents who were prescribed three or more (Table 3). Figure 1 outlines the patterns of analgesic and adjuvant medication co‐prescribing in Australian and Finnish samples.

**TABLE 3:** Number of Australian and Finnish residents prescribed one or more regular analgesic and/or adjuvant medications.

Number of analgesics or adjuvant medications within analgesic regimens	Australia (*n* = 416 residents prescribed any analgesic or adjuvant medication) *n* (%)	Finland (*n* = 1406 residents prescribed any analgesic or adjuvant medication) *n* (%)
1 (monotherapy)	235 (57)	937 (67)
2	115 (28)	382 (27)
3	49 (12)	73 (5)
4	15 (4)	14 (1)
5	2 (1)	0 (0)

Residents in the Australian sample had a higher prevalence of co‐prescribing across all analgesic and adjuvant medication combinations, with the exception of NSAIDs and opioids: of eight Australian and 14 Finnish residents prescribed NSAIDs, 3 (38%) vs. 7 (50%) were co‐prescribed opioids. Acetaminophen was the most frequently co‐prescribed medication in both samples, yet higher in the Australian sample (75%–90%) compared to the Finnish sample (43%–56%). Of 61 Australian residents and 186 Finnish residents prescribed gabapentinoids, 38 (62%) vs. 86 (46%) were co‐prescribed opioids. Conversely, of 165 Australian and 609 Finnish residents prescribed opioids, 38 (23%) vs. 86 (14%) were co‐prescribed gabapentinoids. Of residents prescribed adjuvant medications, 59%–83% of Australian and 25%–46% of Finnish residents were co‐prescribed opioids.

## 4 DISCUSSION

This was the first study to explicitly explore analgesic and adjuvant medication co‐prescribing among residents of Australian and Finnish residential care homes. While the general patterns of co‐prescribing were similar in Australian and Finnish residents, co‐prescribing of three or more regular analgesic and/or adjuvant medications was 2.5‐times more prevalent in Australian residents. The higher prevalence of co‐prescribing in Australian residents was largely driven by a high level of acetaminophen co‐prescribing. Further investigation into central nervous system (CNS)‐active polypharmacy is needed in both countries, arising from high rates of opioid co‐prescribing with adjuvant medications.

Our study identified that approximately half (44%) of Australian and one‐third (33%) of Finnish residents were co‐prescribed more than one analgesic and/or adjuvant medication. There was a notable 2.5‐fold higher prevalence of prescribing of three or more analgesic and/or adjuvant medications in Australian residents. Given that Australian and Finnish residents have similar clinical and demographic characteristics, this difference in co‐prescribing was unlikely to fully reflect or explain the apparent differences in pain prevalence.^1^, ^2^, ^3^, ^4^, ^15^, ^16^ The higher rates of co‐prescribing may be partly explained by Australian initiatives to minimise un‐ or under‐managed pain in residential care homes as a possible cause of behavioural symptoms.^25^, ^26^ In the literature, an increase in the use of analgesic medications has been identified as one of the top five factors contributing to the increasing polypharmacy in Australian residential care homes.^27^ Previous research has suggested that analgesic decision‐making varies between countries and cultures.^28^ This highlights the importance of further cross‐national exchanges of knowledge, practice and perspectives between countries with ageing populations, particularly towards analgesic decision‐making. These cross‐national comparative studies are essential for the development of targeted interventions aimed at improving the safe and effective use of these medications in residential care homes.

Acetaminophen was the most frequently co‐prescribed medication, with three‐quarters of Australian residents and over one‐third of Finnish residents prescribed NSAIDs, opioids, gabapentinoids, TCAs or duloxetine, co‐prescribed acetaminophen. Acetaminophen is considered the first‐line analgesic for mild‐to‐moderate pain in older adults and may potentiate the effects of opioids, minimising the need for higher doses of opioids and adjuvant medications, hence reducing ADEs.^6^, ^29^ We were not able to investigate whether acetaminophen was co‐prescribed to minimise opioid doses (i.e. opioid sparing) in this study, and further research is needed to explore this practice. Our findings suggest that Australian clinicians continued regular acetaminophen when acetaminophen alone was not achieving the required level of analgesia. The efficacy of acetaminophen in the context of chronic non‐cancer pain in older adults has recently been scrutinised.^29^ The *British Journal of Pain* (2022), European Society for Clinical and Economic Aspects of Osteoporosis (ESCEO, 2019), National Institute for Health and Care Excellence (NICE, 2021) and the Australian National Prescribing Service (NPS, 2022) have highlighted the limited evidence of acetaminophen efficacy for chronic pain and potential ADEs with long‐term use, particularly when co‐prescribed with NSAIDs.^30^, ^31^, ^32^, ^33^ Favourably, the co‐prescribing of acetaminophen with NSAIDs was close to zero per cent across all residents in our study. Further understanding of the long‐term safety and efficacy of acetaminophen within older adults' analgesic regimens is needed.

Approximately three in five Australian residents and two in five Finnish residents prescribed gabapentinoids were co‐prescribed opioids. The prevalence of gabapentinoid prescribing in residential care homes has reportedly increased in Finland (1% in 2003 to 9% in 2017), Ontario (2% in 2010 to 12% in 2019) and Norway (1% in 2000 to 4% in 2011).^18^, ^34^, ^35^ Specifically, the prevalence of co‐prescribed gabapentinoids with opioids in the United States was 48% higher in 2018 than in 2011.^36^ The rising use of gabapentinoids among older adults, alone and in combination with opioids, has sparked safety concerns due to increased risks of CNS‐active polypharmacy, which is associated with heightened likelihood of sedation, cognitive impairment, falls and other serious adverse drug events.^37^ This concern was reflected in the American Geriatrics Society (AGS) Beers Criteria (2019, updated in 2023), which strongly recommends against the co‐prescribing of opioids and gabapentinoids in older adults.^38^ A recent Australian study reported gabapentinoid dispensing was associated with a higher risk of hip fractures, especially in frail individuals, which persisted after considering concomitant use of other CNS medications.^39^ Our study highlights the importance of comprehensive, holistic interventions that consider a resident's entire medication regimen, including combinations that increase susceptibility of analgesic‐related ADEs, such as the co‐prescribing of CNS‐active medications with opioids.

Over 59% of Australian residents who used adjuvant medications were co‐prescribed opioids, compared to 25% of Finnish residents. Adjuvant medications are recommended for alleviating certain types of neuropathic pain, yet frequently prescribed for non‐neuropathic pain.^40^ Though adjuvant medications provide a potentially safer alternative to opioids for pain management in the perioperative setting, the safety of adjuvants for chronic pain in older adults is not well established.^41^, ^42^ Nevertheless, our study identified that opioid and adjuvant medication co‐prescribing was common in Australian and Finnish residential care homes. The value of system‐level monitoring and analgesic stewardship interventions in residential care homes has been gaining international recognition in recent years.^43^, ^44^ Analgesic indicators have been identified as a possible mechanism for residential care homes to monitor safe and effective analgesic use, ensure effective pain management and reduce analgesic‐related harm.^45^, ^46^ An indicator of residents prescribed opioids with other CNS‐active medications has been proposed to identify residents who may benefit from multidisciplinary medication reviews and introduction of non‐pharmacological approaches.^45^, ^47^ Advances in electronic medication management systems represent a unique opportunity to efficiently implement indicators and reform analgesic optimisation in residential care homes.

The lower utilisation of adjuvant medications in Finland, including a 12‐fold lower prevalence of TCAs, may be partly explained by Finnish‐specific guidelines recommending avoidance of TCAs in older adults due to their anticholinergic effects.^48^ Variations in adjuvant medication prevalence may partly reflect differences in cultural attitudes or clinical guidelines for managing non‐pain conditions, such as depression, rather than pain management practices between the two countries.^28^ The Meds75+ database (the national Finnish criteria used to support clinical decision‐making regarding drug treatment for people greater than 75 years of age) lists TCAs as a medication to avoid in older adults due to significant ADEs (category D) and gabapentinoids and duloxetine as more suitable alternatives (category C).^49^ This may explain the preference towards duloxetine in the Finnish sample.

### 4.1 Strengths and limitations

A key strength of this study was that residents who participated were representative of all residents living in nursing homes or assisted living facilities residential care homes in Helsinki. The co‐prescribing matrices used in this study represent a novel approach to exploring and reporting analgesic and adjuvant utilisation in residential care homes. This study contributes to a high‐priority research area in residential care homes.^9^, ^10^, ^11^

Australian and Finnish residents included in this study were comparable in terms of age and sex. Pain assessment measurements between the two samples were not directly comparable, as the prevalence and intensity of pain varies according to the pain assessment method used (i.e. observational or self‐report measures).^50^ Future comparative studies may explore analgesic and adjuvant prescribing practices based on pain control, using the same proxy measure for self‐reported and observed pain prevalence. The 15D ‘discomfort and pain’ item used to report pain in the Finnish sample includes symptoms of pain, ache, nausea and itching, which may have overestimated actual pain prevalence.

The Finnish sample excluded residents with moderate‐to‐severe dementia, whereas the Australian sample excluded residents considered to be at the end of life (<3 months to live) and those not able to complete baseline assessments, which may partly explain differences in prescribing. A 2015 systematic review with meta‐analyses reported that residents living with dementia or cognitive impairment had a significantly lower prevalence of analgesic use compared to residents without cognitive impairment (odds ratio [OR] .6, 95% confidence interval [CI] .4–.8).^50^ However, previous research with the same aged care provider in Australia reported similar analgesic prevalence in residents with and without dementia (79% vs. 73.4%, *p* = .20).^4^ Further research exploring analgesic and adjuvant co‐prescribing patterns based on cognitive impairment is required.

This study explored prescribing of regular medications, which may underestimate true usage, as acetaminophen and opioids are often listed as a PRN medication and administered at the discretion of registered nurses.^26^, ^28^, ^45^

Data were derived from South Australia and Helsinki and therefore, the results may not be generalisable to overall prescribing patterns in each country. Differences in the number of residents included in the Australian (*n* = 550) and Finnish (*n* = 2423) samples should be considered when interpreting the results. This study involved secondary analysis of data. There was limited scope to explore differences in non‐pharmacological approaches, topical NSAIDs, prescribed doses of medications or indications. This is important because adjuvant medications may be used for non‐pain indications (e.g. depression). For these reasons, it was not possible to comment on the clinical appropriateness of medication regimens for pain. However, pharmacological interactions between analgesic and adjuvant medications remain an important consideration regardless of the prescribing indication.

## 5 CONCLUSIONS

Analgesic and adjuvant co‐prescribing was more prevalent for Australian than Finnish residents, particularly co‐prescribing of three or more analgesic and/or adjuvant medications. This was largely driven by acetaminophen co‐prescribing to more than three‐quarters of analgesic or adjuvant users in Australia. CNS‐active polypharmacy arising from high rates of opioid and adjuvant co‐prescribing warrants further attention in both countries. Cross‐national research and targeted analgesic stewardship interventions are needed to ensure the safe and effective co‐prescribing of analgesic and adjuvant medications in residential care homes.

## FUNDING INFORMATION

The Frailty in Residential Sector over Time (FIRST) Study and ADJ were funded through project funding to RV from the Healthy Aging Research Consortium funded by the South Australian Department for Innovation and Skills, Hospital Research Foundation and Resthaven Inc. LAD and SJL were supported by postgraduate research scholarships funded by Monash University. SJL, RV, and JSB were supported by the NHMRC Centre of Research Excellence in Frailty and Healthy Ageing. SJL was supported by the Australian Government Research Training Program Scholarship. AJC is supported by an NHMRC Emerging Leadership 1 grant (APP2009633).

## CONFLICT OF INTEREST STATEMENT

RV was previously on the board of Resthaven and the board governance committee. Visvanathan is co‐founder and chair of the clinical advisory group for a wearable sensor technology start‐up HealthVibes.ai. AJC has received grant or consulting funds from the Medical Research Future Fund and the Pharmaceutical Society of Australia. All these funds were paid to the administering University. AJC is also a national board director for the Pharmaceutical Society of Australia. JSB has received grant or consulting funds from the NHMRC, Medical Research Future Fund, Victorian Government Department of Health and Human Services, Dementia Australia Research Foundation, Yulgilbar Foundation, Aged Care Quality and Safety Commission, Australian Commission on Safety and Quality in Health Care, Dementia Centre for Research Collaboration, Pharmaceutical Society of Australia, Society of Hospital Pharmacists of Australia, GlaxoSmithKline Supported Studies Programme, Amgen, and several aged care provider organisations. All these funds were paid to the administering University. All other authors have no conflicts of interest to declare.

## Supporting information


Files S1–S2

## Data Availability

The data that support the findings of this study are available from the corresponding author upon reasonable request.
