# Supplementary material for: Analgesic and adjuvant co‐prescribing in Australian and Finnish residential care homes
Source: Australas J Ageing. 2025 Jul 1;44(3):e70062. doi: 10.1111/ajag.70062 (PMC12210788; doi:10.1111/ajag.70062)
Supplement: Supplementary file 1 — Files S1–S2 [file AJAG-44-0-s001.docx]

**Supplementary file 1:** Classification of analgesics using the ATC Classification System recommended by the WHO^24^

| **Analgesics and adjuvant medications** | | | **Specific ATC codes** |
| --- | --- | --- | --- |
| Salicylic acid derivatives (**N02BE01**) or acetaminophen combinations, excluding psycholeptics (**N02BE51**) | Acetaminophen | Acetaminophen | N02BE01 |
|  | Acetaminophen + non-opioid | Acetaminophen + ibuprofen | N02BE51 |
| Anti-inflammatory and antirheumatic products, non-steroids (**M01A**) | NSAIDs | Ibuprofen | M01AE01 |
|  |  | Diclofenac | M01AB05 |
|  |  | Meloxicam | M01AC06 |
|  |  | Naproxen | M01AE02 |
|  |  | Mefenamic acid | M01AG01 |
|  |  | COX-2 inhibitors | |
|  |  | Celecoxib | M01AH01 |
| Opioids (**N02A**), codeine (**R05DA04**) or opioids in combination with non-opioid analgesics (**N02AJ**) | Opioids | Weak | |
|  |  | Codeine | R05DA04 |
|  |  | Dihydrocodeine | N02AA58 |
|  |  | Tapentadol | N02AX06 |
|  |  | Tramadol | N02AX02 |
|  |  | Strong | |
|  |  | Buprenorphine | N02AE01 |
|  |  | Fentanyl | N02AB03 |
|  |  | Hydromorphone | N02AA03 |
|  |  | Morphine | N02AA01 |
|  |  | Oxycodone | N02AA05 |
|  |  | Oxycodone + Naloxone | N02AA55 |
|  |  | Oxymorphone | N02AA11 |
|  | Opioids + non-opioid | Dihydrocodeine + acetaminophen | N02AJ01 |
|  |  | Dihydrocodeine + acetylsalicylic acid | N02AJ02 |
|  |  | Dihydrocodeine + other non-opioid analgesics | N02AJ03 |
|  |  | Codeine + acetaminophen | N02AJ06 |
|  |  | Codeine + acetylsalicylic acid | N02AJ07 |
|  |  | Codeine + ibuprofen | N02AJ08 |
|  |  | Codeine + other non-opioid analgesics | N02AJ09 |
|  |  | Tramadol + acetaminophen | N02AJ13 |
|  |  | Tramadol + other non-opioid analgesics | N02AJ15 |
|  |  | Tramadol + celecoxib | N02AJ16 |
| Gabapentinoids (**N02BF**) | Gabapentinoids | Gabapentin | N02BF01 |
|  |  | Pregabalin | N02BF02 |
| Non-selective monoamine reuptake inhibitors (**N06AA**) | TCAs | Amitriptyline | N06AA09 |
|  |  | Nortriptyline | N06AA10 |
|  |  | Dosulepin/dothiepin | N06AA16 |
|  |  | Doxepin | N06AA12 |
|  |  | Imipramine | N06AA02 |
| Other antidepressants (**N06AX**) | SNRIs | Duloxetine | N06AX21 |

**Supplementary file 2:** Master data collection table: Prevalence of co-prescribing of traditional analgesics and adjuvant medications

Australian sample (n=550)

| **Medications** | **ATC codes** | **Regular, n** |
| --- | --- | --- |
| Acetaminophen (total) | N02BE01 | 380 |
| Acetaminophen + NSAID | N02BE01 + M01A or N02BE51^*^ | 6 |
| Acetaminophen + Opioid | N02BE01 + (N02A, R05DA04) or N02AJ^†^ | 136 |
| Acetaminophen + Gabapentinoid | N02BE01 + N02BF | 55 |
| Acetaminophen + TCA | N02BE01 + N06AA | 28 |
| Acetaminophen + Duloxetine | N02BE01 + N06AX21 | 9 |
| NSAID (total) | M01A | 8 |
| NSAID + Opioid | M01A + (N02A, R05DA04) | 3 |
| NSAID + Gabapentinoid | M01A + N02BF | 1 |
| NSAID + TCA | M01A + N06AA | 0 |
| NSAID + Duloxetine | M01A + N06AX21 | 0 |
| Opioids (total) | (N02A, R05DA04) | 165 |
| Opioid + Gabapentinoid | (N02A, R05DA04) + N02BF | 38 |
| Opioid + TCA | (N02A, R05DA04) + N06AA | 20 |
| Opioid + Duloxetine | (N02A, R05DA04) + N06AX21 | 10 |
| Gabapentinoid (total) | N02BF | 61 |
| Gabapentinoid + TCA | N02BF + N06AA | 8 |
| Gabapentinoid + Duloxetine | N02BF + N06AX21 | 6 |
| TCA (total) | N06AA | 34 |
| TCA + Duloxetine | N06AA + N06AX21 | 1 |
| Duloxetine (total) | N06AX21 | 12 |

Finnish sample (n=2,423)

| **Medications** | **ATC codes** | **Regular, n** |
| --- | --- | --- |
| Acetaminophen (total) | N02BE01 | 1,070 |
| Acetaminophen + NSAID | N02BE01 + M01A or N02BE51^*^ | 6 |
| Acetaminophen + Opioid | N02BE01 + (N02A, R05DA04) or N02AJ^†^ | 341 |
| Acetaminophen + Gabapentinoid | N02BE01 + N02BF | 104 |
| Acetaminophen + TCA | N02BE01 + N06AA | 6 |
| Acetaminophen + Duloxetine | N02BE01 + N06AX21 | 52 |
| NSAID (total) | M01A | 14 |
| NSAID + Opioid | M01A + (N02A, R05DA04) | 7 |
| NSAID + Gabapentinoid | M01A + N02BF | 0 |
| NSAID + TCA | M01A + N06AA | 0 |
| NSAID + Duloxetine | M01A + N06AX21 | 0 |
| Opioids (total) | (N02A, R05DA04) | 609 |
| Opioid + Gabapentinoid | (N02A, R05DA04) + N02BF | 86 |
| Opioid + TCA | (N02A, R05DA04) + N06AA | 3 |
| Opioid + Duloxetine | (N02A, R05DA04) + N06AX21 | 32 |
| Gabapentinoid (total) | N02BF | 186 |
| Gabapentinoid + TCA | N02BF + N06AA | 2 |
| Gabapentinoid + Duloxetine | N02BF + N06AX21 | 26 |
| TCA (total) | N06AA | 12 |
| TCA + Duloxetine | N06AA + N06AX21 | 0 |
| Duloxetine (total) | N06AX21 | 95 |

NSAIDs, Nonsteroidal Anti-inflammatory Drugs; PRN, Pro Re Nata; TCAs, Tricyclic Antidepressants.

^*^N02BE51= products including acetaminophen in combination with ibuprofen

^†^N02AJ= products including opioids in combination with non-opioid analgesics. Examples: dihydrocodeine + acetaminophen (N02AJ01), dihydrocodeine + acetylsalicylic acid (N02AJ02), dihydrocodeine + other non-opioid analgesics (N02AJ03), codeine + acetaminophen (N02AJ06), codeine + acetylsalicylic acid (N02AJ07), codeine + ibuprofen (N02AJ08), codeine + other non-opioid analgesics (N02AJ09), tramadol + acetaminophen (N02AJ13), tramadol + other non-opioid analgesics (N02AJ15) or tramadol + celecoxib (N02AJ16).
